# Polyethylenimine-Crosslinked 3-Aminopropyltriethoxysilane-Grafted Multiwall Carbon Nanotubes for Efficient Adsorption of Reactive Yellow 2 from Water

**DOI:** 10.3390/ijms24032954

**Published:** 2023-02-03

**Authors:** Zhuo Wang, Sung Wook Won

**Affiliations:** 1 Department of Ocean System Engineering, Gyeongsang National University, 2 Tongyeonghaean-ro, Tongyeong 53064, Gyeongnam, Republic of Korea; 2 Department of Marine Environmental Engineering, Gyeongsang National University, 2 Tongyeonghaean-ro, Tongyeong 53064, Gyeongnam, Republic of Korea

**Keywords:** amine functionalized MWCNTs, reactive yellow 2, adsorption, rapid removal, ionic strength, reusability

## Abstract

This research intended to report amine-functionalized multiwall carbon nanotubes (MWCNTs) prepared by a simple method for efficient and rapid removal of Reactive Yellow 2 (RY2) from water. EDS analysis showed that the N content increased from 0 to 2.42% and from 2.42 to 8.66% after modification by 3-Aminopropyltriethoxysilane (APTES) and polyethylenimine (PEI), respectively. BET analysis displayed that the specific surface area, average pore size, and total pore volume were reduced from 405.22 to 176.16 m^2^/g, 39.67 to 6.30 nm, and 4.02 to 0.28 cm^3^/g, respectively. These results proved that the PEI/APTES-MWCNTs were successfully prepared. pH edge experiments indicated that pH 2 was optimal for RY2 removal. At pH 2 and 25 °C, the time required for adsorption equilibrium was 10, 15, and 180 min at initial concentrations of 50, 100, and 200 mg/L, respectively; and the maximum RY2 uptake calculated by the Langmuir model was 714.29 mg/g. Thermodynamic studies revealed that the adsorption process was spontaneous and endothermic. Moreover, 0–0.1 mol/L of NaCl showed negligible effect on RY2 removal by PEI/APTES-MWCNTs. Five adsorption/desorption cycles confirmed the good reusability of PEI/APTES-MWCNTs in RY2 removal. Overall, the PEI/APTES-MWCNTs are a potential and efficient adsorbent for reactive dye wastewater treatment.

## 1. Introduction

It is estimated that around 4 billion people worldwide face short- or long-term water scarcity [1]. Moreover, by 2050, this population will grow to approximately 6 billion, which may even be underestimated [2]. Water scarcity can lead to various social and environmental problems and, ultimately, to war [3,4]. The textile industry is one of the industries that exacerbates water scarcity. On the one hand, the textile industry is water-consuming, and in contrast, its wastewater discharge is enormous. The immediate release of untreated textile wastewater can cause severe pollution of water sources [5]. Reactive dyes are counted among the most frequently used dyes in the dyeing industry. The typical characteristics of their wastewater include difficult biodegradation, high coloration even at low concentrations, and high organic concentrations [6]. Reactive dyes and their degradation by-products are toxic, carcinogenic, teratogenic, and mutagenic [7]. Many techniques have been developed by researchers to eliminate dyes from wastewater, including coagulation, flocculation, biological processes, membrane separation, microextraction, and photocatalysis [8,9]. Some disadvantages of these methods are their high cost, low efficiency, formation of toxic by-products, and generation of large amounts of concentrated sludge [10]. Because of these limitations, simple, inexpensive, and effective methods are needed to remove dyes from wastewater. Adsorption is considered one of the most desirable methods for dye removal due to its low cost, simplicity of operation, low energy requirements, and recoverable adsorbent [10,11].

In recent decades, nanomaterials have attracted much attention due to their particular structures and unique physicochemical properties. Nanomaterials, including metal oxides, carbon nanotubes, graphene nanosheets, silicon nanospheres, and polymeric nanofibers, have been used as adsorbents to reduce various pollutants from wastewater [12,13]. Among them, multiwall carbon nanotubes (MWCNTs) are considered excellent adsorbents for removing organic dyes from water because of their large specific surface area, easy surface modification, and good chemical stability [14,15]. Since the adsorption capacity of pristine MWCNTs for organic dyes was relatively low, many researchers have used surface modification methods, such as amine-functionalization methods, to improve their adsorption capacity [15]. However, the preparation of amine-functionalized MWCNTs often involves the utilization of toxic organic solvents, extreme conditions, and complex procedures [16,17,18,19]. Therefore, it is still of great significance to explore more accessible and greener ways to prepare amine-functionalized MWCNTs.

In this study, amine-functionalized MWCNTs, namely, polyethylenimine (PEI)-crosslinked 3-aminopropyltriethoxysilane (APTES)-grafted MWCNTs (PEI/APTES-MWCNTs), were prepared in a more straightforward and organic solvent-free approach. The adsorption of reactive dyes by amine-functionalized MWCNTs has yet to be reported. Reactive Yellow 2 (RY2), a widely used reactive dye, was selected as a representative to evaluate the adsorption performance of reactive dyes on PEI/APTES-MWCNTs. The effects of pH, contact time, initial concentration, temperature, and ionic strength were estimated to understand the adsorption performance of PEI/APTES-MWCNTs towards RY2. The reusability of the adsorbent was also investigated.

## 2. Results and Discussion

**Figure 1:**
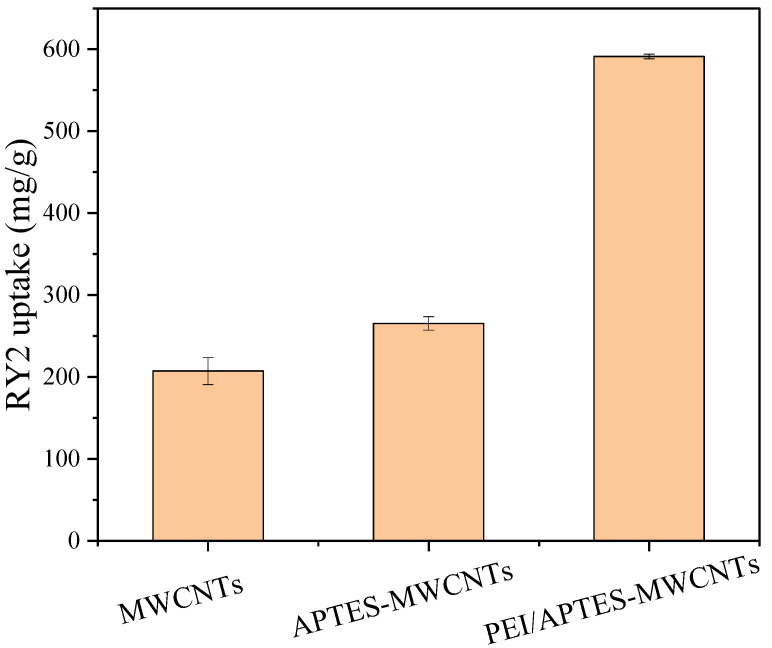
RY2 uptake on MWCNTs, APTES-MWCNTs, and PEI/APTES-MWCNTs. (C_i_ = 200 mg/L, sorbent amount = 10 mg, pH = 2).

In this work, PEI/APTES-MWCNTs were prepared by a simple and feasible method. The one-point-check experiment was performed to verify the successful preparation of PEI/APTES-MWCNTs. As shown in Figure 1, the adsorption capacity of MWCNTs was increased 1.3 times after grafting APTES on their surface. After crosslinking with PEI, the adsorption capacity increased to about 2.8 times that of the pristine MWCNTs. This result proved that the PEI/APTES-MWCNTs were successfully prepared. FE-SEM-EDS and BET analyses were performed to investigate the prepared adsorbents further.

### 2.1. FE-SEM-EDS Analysis

**Figure 2:**
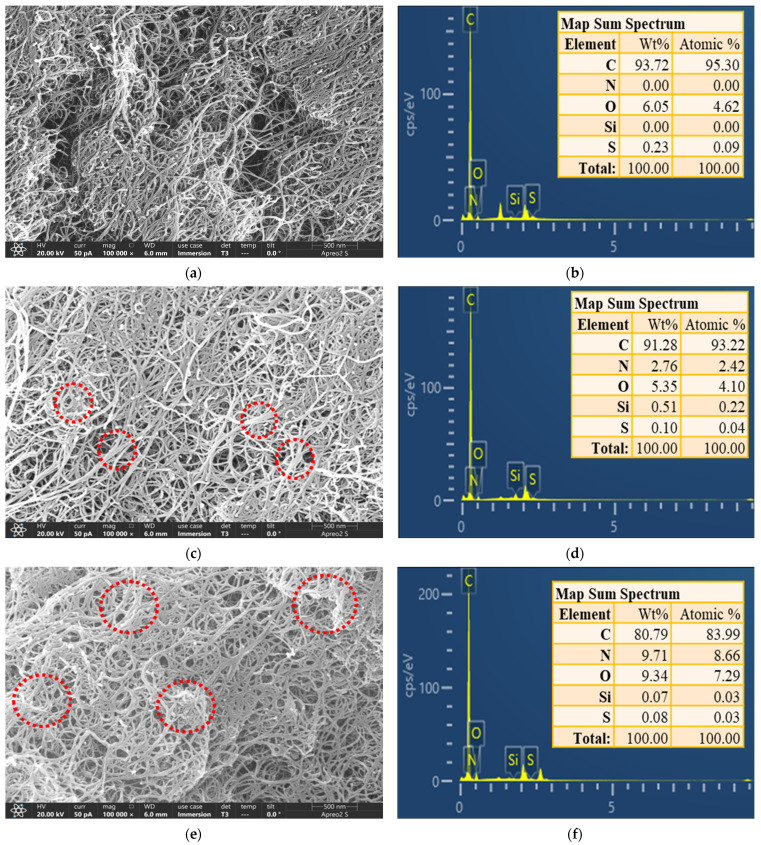
FE-SEM image (magnification ×100,000) and map sum spectrum of (**a**,**b**) MWCNTs, (**c**,**d**) APTES-MWCNTs, and (**e**,**f**) PEI/APTES-MWCNTs.

The surface morphology of the materials was observed using a field-emission scanning electron microscope (FE-SEM), and their corresponding elemental mappings were recorded using an energy-dispersive spectrometer (EDS). As shown in Figure 2a,c, the spacing between the APTES-grafted MWCNTs was lower than that of the original MWCNTs. In addition, numerous MWCNTs aggregated into bundles, as shown in the red circles in Figure 2c. The interval between MWCNTs after PEI crosslinking was smaller, and more MWCNTs were entangled, as shown in the red circle (Figure 2e). This higher entangled structure may be due to the functionalization of MWCNTs [18]. The aggregation structure may be due to the enhanced interactions between functionalized MWCNTs [20]. The PEI/APTES-MWCNTs show severe entanglement compared to the pristine MWCNTs and APTES-MWCNTs. The reason was that the PEI molecules crosslinked the MWCNTs that were originally present in a dispersed state. The FE-SEM images may confirm that APTES and PEI successfully modified the MWCNTs. To further verify this conclusion, the EDS analysis was conducted. Figure 2b revealed that the MWCNTs did not contain N and Si elements. As demonstrated in Figure 2d, N and Si elements appeared in the map sum spectrum of APTES-MWCNTs, indicating that APTES was grafted on the MWCNTs’ surface. With further modification with PEI, the N elemental content in functionalized MWCNTs was greatly increased (Figure 2f). The atomic percentage of N in PEI/APTES-MWCNTs was approximately 3.6 times higher than in APTES-MWCNTs. Overall, FE-SEM-EDS analysis proved that APTES and PEI successfully modified MWCNTs.

### 2.2. FTIR Analysis

**Figure 3:**
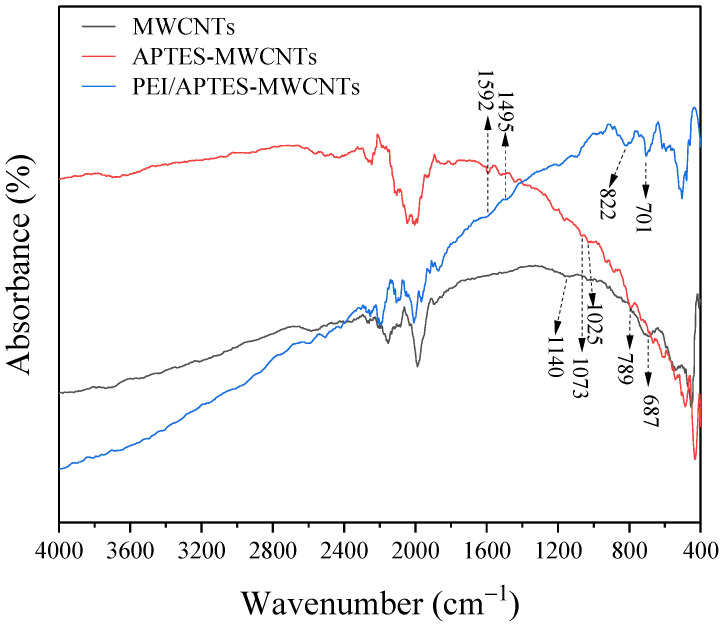
FTIR spectra of MWCNTs, APTES-MWCNTs, and PEI/APTES-MWCNTs.

The FTIR spectra of MWCNTs, APTES-MWCNTs, and PEI/APTES-MWCNTs are compared in Figure 3. The presence of the broadband between 3400 and 3800 and 1140 cm^−1^ is due to the presence of hydroxyl groups on the surface of MWCNTs, which was caused by moisture in the ambient air or by oxidation of raw materials during the purification process [21]. The peak at 687 cm^−1^ was attributed to aromatic C–H bending vibration [22]. After being grafted by APTES, new peaks appeared in the FTIR spectrum of APTES-MWCNTs. The peak at 1592 cm^−1^ was assigned to –NH_2_ bending vibration [23]. The weak peaks displayed at 1073 and 1025 cm^−1^ were attributed to C–O–Si and Si–O–Si stretching vibrations [24], while the peak at 789 cm^−1^ was due to Si–O–Si symmetric stretching vibrations [25]. These results revealed that the APTES molecules were successfully grafted on the surface of MWCNTs. In the FTIR spectrum of PEI/APTES-MWCNTs, the peaks at 1592 and 1495 cm^−1^ were attributed to N–H bending vibrations of –NH_2_ and –NH, respectively [23,26]. In addition, the peaks at 822 and 701 cm^−1^ were assigned to C–N outside rocking vibration [26]. The above results suggested that PEI molecules were crosslinked with APTES-MWCNTs.

### 2.3. BET Analysis

**Figure 4:**
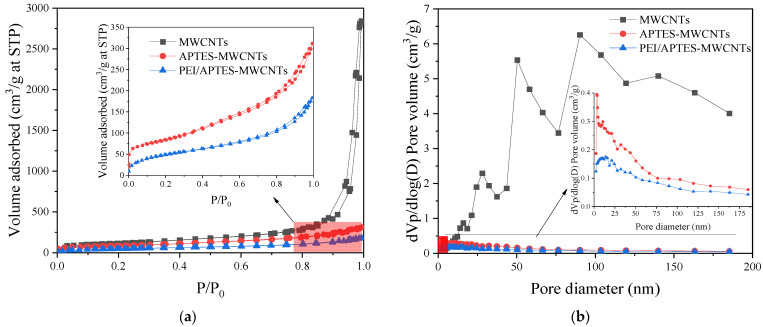
(**a**) N_2_ adsorption–desorption isotherms and (**b**) pore size distribution of MWCNTs, APTES-MWCNTs, and PEI/APTES-MWCNTs.

The specific surface area, average pore size, and total pore volume of MWCNTs, APTES-MWCNTs, and PEI/APTES-MWCNTs were measured by N_2_ adsorption-desorption experiments; the results are presented in Figure 4 and Table 1, respectively. According to the IUPAC classification [27], the curve of MWCNTs is type IV isotherm with a type H1 hysteresis loop, while the curves of APTES-MWCNTs and PEI/APTES-MWCNTs are type IV isotherm with a type H3 hysteresis loop (Figure 4a). These results indicate the presence of mesopores [28,29]. The pore size distributions of the samples are shown in Figure 4b. It can be seen that MWCNTs have large pore volumes, with many of their pore sizes exceeding 15 nm (the diameter of the commercial MWCNTs is 8–15 nm); this should be associated with the secondary apertures created by the aggregation of MWCNTs [30]. The pore volumes and the number of large pores (>15 nm) of MWCNTs were significantly reduced due to the modification by APTES and PEI. These results were probably attributed to APTES and PEI blocking of the pores and interspace regions between MWCNTs. As a result, the specific surface area, average pore size, and total pore volume of APTES-MWCNTs and PEI/APTES-MWCNTs were much smaller than those of the MWCNTs (Table 1). Although the specific surface area and total pore volume of PEI/APTES-MWCNTs were much smaller than those of MWCNTs, their ability to adsorb RY2 was greatly enhanced (Figure 1). The reduction in specific surface area and total pore volume was attributed to the large number of adsorption sites provided by the amine groups of PEI, indicating that PEI was successfully crosslinked to the surface of APTES-MWCNTs.

### 2.4. Effect of pH

**Figure 5:**
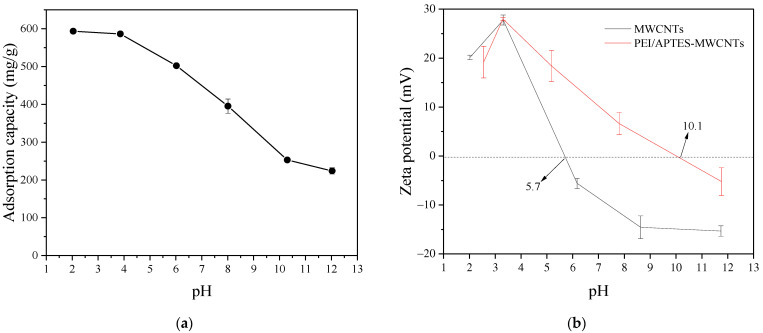
(**a**) Effect of pH on RY2 adsorption on PEI/APTES-MWCNTs and (**b**) zeta potential of MWCNTs and PEI/APTES-MWCNTs at different pHs.

Solution pH is an important factor affecting the adsorption performance of adsorbents. The relationship between the solution pH and the adsorption capacity of RY2 on PEI/APTES-MWCNTs was investigated by altering the initial pH of the RY2 solution under controlled experimental conditions (Figure 5a). The PEI/APTES-MWCNTs showed the highest adsorption capacity (593.6 ± 1.3 mg/g) for RY2 at pH 2.0. The dye uptake on PEI/APTES-MWCNTs slightly decreased to 586.4 ± 3.8 mg/g with increasing pH to 4.0. Hereafter, the adsorption capacity of PEI/APTES-MWCNTs for RY2 dropped sharply to 224.0 ± 8.3 mg/g when the pH was raised to 12.0. To further explain the adsorption performance of PEI/APTES-MWCNTs for RY2, zeta potential analysis was performed, and the results are displayed in Figure 5b. The pH_IEP_ (isoelectric point of pH) of the MWCNTs increased from 5.7 to 10.1 after modification by APTES and PEI, indicating that APTES and PEI successfully modified the MWCNTs. In addition, the surface of PEI/APTES-MWCNTs is positively charged when the solution pH is below 10.1 and negatively charged when the pH is above 10.1. On the other hand, the *pK_a_* of primary, secondary, and tertiary amine groups are 4.5, 6.7, and 11.6, respectively [31]. Thus, at pH < pH_IEP_, the primary and secondary amine groups on PEI/APTES-MWCNTs surface were protonated. The protonated primary (NH_3_^+^) and secondary (NH_2_^+^) amine groups readily bind to the negatively charged sulfonate groups (-SO_3_^2−^) of RY2 via electrostatic interaction [32]. On the contrary, the gradual deprotonation of protonated amine groups with the increase in pH reduced RY2 uptake [33]. When pH > pH_IEP_, the surface of PEI/APTES-MWCNTs was entirely deprotonated, but the uptake of RY2 was still evident. In this case, the adsorption of RY2 on PEI/APTES-MWCNTs was mainly achieved by π–π stacking and hydrogen bonding [19,34]. Since the maximum dye uptake was reached at pH 2, it was chosen for the subsequent studies.

### 2.5. Adsorption Studies

**Figure 6:**
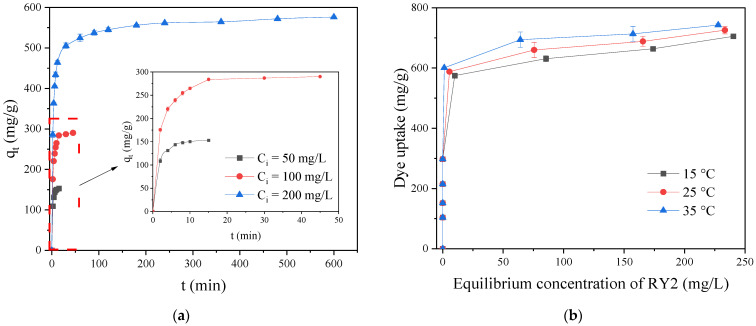
(**a**) Adsorption kinetics of RY2 on PEI/APTES-MWCNTs at 50–200 mg/L initial concentrations; (**b**) adsorption isotherm of RY2 on PEI/APTES-MWCNTs at different temperatures (15–35 °C).

Adsorption time, initial solute concentration, and solution temperature are leading factors that influence the adsorption performance of adsorbents. The effects of contact time, initial RY2 concentration, and temperature on RY2 adsorption onto PEI/APTES-MWCNTs were investigated, and the results are shown in Figure 6. Figure 6a displays the effect of contact time on the adsorption of RY2 onto PEI/APTES-MWCNTs at different initial concentrations. The time required to reach adsorption equilibrium was very short at low concentrations and pretty long at high concentrations. The time required for adsorption equilibrium was about 10, 15, and 180 min at initial concentrations of 50, 100, and 200 mg/L, respectively. It is worth noting that despite the relatively long time required to reach adsorption equilibrium at high concentrations (200 mg/L), 80% of its maximum adsorption capacity was attained within 30 min, which is of great interest in practical applications. Figure 6b demonstrates the influence of the initial RY2 concentration on its adsorption on PEI/APTES-MWCNTs, together with the influence of solution temperature as evaluated by varying the temperature from 15 to 35 °C. It can be noticed that the slope of the curve is very steep at the initial stage, indicating that the removal of dyestuff at low concentrations was very high. The experimental results revealed that the removal rate of dyes in the initial concentration range of 30–200 mg/L was greater than 95%. Moreover, as the temperature increased, the maximum adsorption capacity rose, indicating that the adsorption process was endothermic. For a further understanding of the adsorption performance, kinetics, isotherm, and thermodynamic models were used to analyze the experimental data.

#### 2.5.1. Adsorption Kinetics

The adsorption rates, possible adsorption mechanisms, and potential rate-controlling steps of RY2 adsorption on PEI/APTES-MWCNTs were studied using pseudo-first-order (PFO), pseudo-second-order (PSO), and intraparticle diffusion (IPD) models. The linearized equations of the PFO [35], PSO [19], and IPD [36] models are as follows:

(1) $$\mathrm{PFO}\;\mathrm{model}:\;\ln\left(q_{e}-q_{t}\right)=\ln q_{e}-k_{1}t$$

(2) $$\mathrm{PSO}\;\mathrm{model}:\;\frac{t}{q_{t}}=\frac{1}{k_{2}q_{e}^{2}}+\frac{t}{q_{e}}$$

(3) $$\mathrm{IPD}\;\mathrm{model}:\;q_{t}=k_{i}t^{0.5}+C$$

where *q_t_* and *q_e_* (mg/g) are the dye uptake at time *t* and equilibrium, respectively; *k*_1_ (min^−1^), *k*_2_ (g/(mg·min)), and *k_i_* (mg/(g·min^0.5^)) are the rate constants of the PFO, PSO, and IPD models, separately; and *C* (mg/g) is the intercept, which indicates the boundary layer thickness.

**Figure 7:**
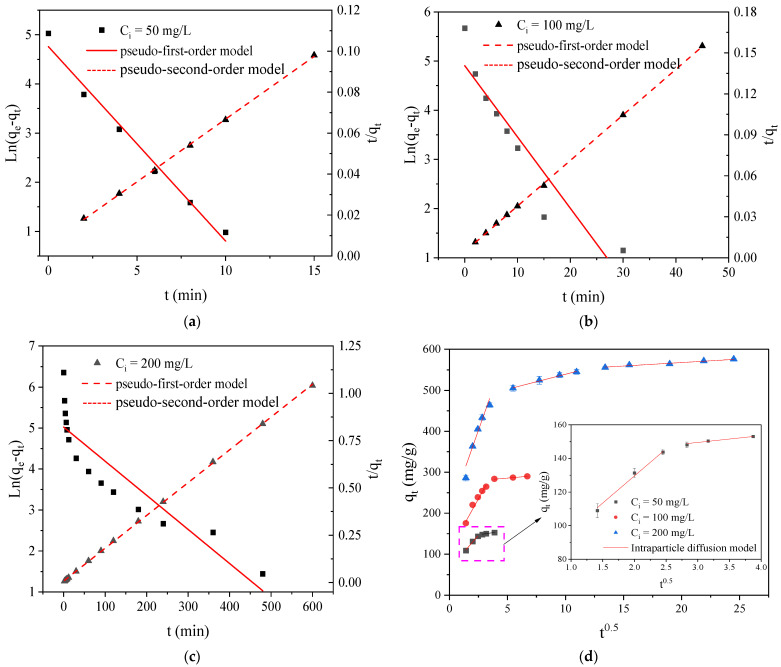
Pseudo-first/second-order modeling of RY2 adsorption on PEI/APTES-MWCNTs at an initial concentration of (**a**) 50, (**b**) 100, and (**c**) 200 mg/L, respectively; (**d**) intraparticle diffusion modeling of RY2 adsorption on PEI/APTES-MWCNTs.

The curves fitted by the PFO and PSO models are displayed in Figure 7a–c, and the corresponding parameters are listed in Table 2. The value of the PSO model’s correlation coefficient (*Adj. R*^2^) was higher than that of the PFO model. Besides, the dye uptake at adsorption equilibrium calculated by the PSO model was closer to that of the experimental data (*q_e,exp_*). These facts suggested that the PSO model was more suitable to explain the kinetic process of RY2 adsorption on PEI/APTES-MWCNTs, and that chemisorption dominated the adsorption process [32,37]. The rate constant (*k*_2_) value decreased with increasing initial concentrations of RY2, revealing that it was a concentration-dependent adsorption process [38]. The reason was that the total number of binding sites on the adsorbent surface was limited. As the dye concentration increased, excessive dye molecules competed for the limited adsorption sites, reducing the adsorption rate constant [39].

The fitted curves of the IPD model and related parameters are depicted in Figure 7d and Table 2, respectively. According to the IPD model, the entire adsorption process mainly covers three sequences: (I) external surface diffusion, (II) intraparticle diffusion, and (III) adsorption equilibrium [40]. All the curves did not pass through the origin, indicating that the adsorption process was influenced by multiple rate-limiting stages [41]. At the initial RY2 concentrations of 50 and 100 mg/L, the fitted curves were split into two distinct regions, suggesting negligible intraparticle diffusion at low concentrations. On the contrary, the fitted curve was divided into three different regions at 200 mg/L initial concentration, demonstrating that the adsorption of RY2 on PEI/APTES-MWCNTs was controlled by external surface diffusion followed by intraparticle diffusion until adsorption equilibrium was reached [32]. Overall, at low concentrations, RY2 adsorption was scarcely affected by intraparticle diffusion, whereas at high concentrations it dominated the adsorption process. On the other hand, intraparticle diffusion was not the only rate-controlling stage, regardless of the concentration of RY2.

#### 2.5.2. Adsorption Isotherms

To investigate whether adsorption occurs on homogeneous or heterogeneous surfaces, the Langmuir and Freundlich isotherm models were used to describe the experimental data. The linear equations of the Langmuir and Freundlich models are given below [19]:

(4) $$\mathrm{Langmuir}\;\mathrm{model}:\;\frac{C_{e}}{q_{e}}=\frac{1}{K_{L}q_{m}}+\frac{C_{e}}{q_{m}}$$

(5) $$\mathrm{Freundlich}\;\mathrm{model}:\;\ln q_{e}=\ln K_{F}+\frac{1}{n}\ln C_{e}$$

where *q_e_* (mg/g) represents the maximum monolayer dye uptake; *C_e_* (mg/L) and *q_e_* (mg/g) are the concentration and adsorption capacity of RY2 at equilibrium; *K_L_* (L/mg) and *K_F_* ((mg/g)(L/mg)^1/*n*^) are the Langmuir and Freundlich constants; *n* indicates the heterogeneity. The values of *K_L_* and *q_m_* were the slope and intercept of the curve plotted by *C_e_*/*q_e_* versus *C_e_*, while the values of *K_F_* and 1/*n* were calculated by the plot of ln*q_e_* versus ln*C_e_*. To determine the favorability of adsorption, the separation factor (*R_L_*) was also calculated using the following equation [37]:

(6) $$R_{L}=\frac{1}{1+K_{L}C_{i}}$$

where *K_L_* (L/mg) is the Langmuir constant, and *C_i_* (mg/L) is the initial RY2 concentration. The *R_L_* values indicate whether the adsorption process is irreversible (*R_L_* = 0), favorable (0 < *R_L_* < 1), linear (*R_L_* = 1), or unfavorable (*R_L_* > 1).

**Figure 8:**
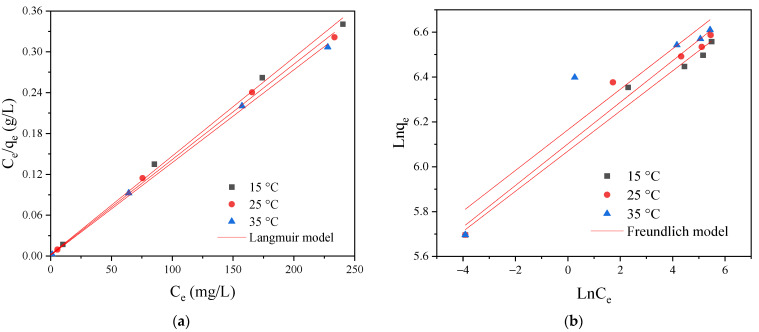
(**a**) Langmuir and (**b**) Freundlich modeling of RY2 adsorption on PEI/APTES-MWCNTs at 15, 25, and 35 °C.

The adsorption isotherm data fitted by the Langmuir and Freundlich models are shown in Figure 8, and the corresponding parameters are listed in Table 3. The higher value of the correlation coefficient (*Adj. R*^2^) of the Langmuir model revealed that it was more applicable to describe the isothermal sorption behavior. The experimental maximum adsorption capacities were close to the theoretical results calculated by the Langmuir model, further supporting its ability to interpret the isothermal data. Therefore, the adsorption of RY2 on PEI/APTES-MWCNTs could be attributed to monolayer adsorption on the homogeneous surface [42]. The Langmuir constant increased with temperature, indicating that the adsorption affinity of the adsorbent for RY2 was higher at high temperatures. The value of the separation factor was between 0 and 1, which was closer to 0, indicating that the adsorption of RY2 on PEI/APTES-MWCNTs was highly favorable. The theoretical maximum dye uptake was 689.66, 714.29, and 735.30 mg/g at 15, 25, and 35 °C, respectively, which were higher than most of the functionalized MWCNTs reported to date (Table 4). The maximum adsorption increased with temperature, indicating the adsorption process was endothermic. Thermodynamic analysis was performed to illustrate further the effect of temperature on the adsorption of RY2 by PEI/APTES-MWCNTs.

#### 2.5.3. Thermodynamic Analysis

Temperature is a critical factor in the adsorption process. The effect of temperature on the adsorption of RY2 by PEI/APTES-MWCNTs is shown in Figure 6b. Thermodynamic parameters were calculated to determine the spontaneity of the adsorption activity, energy changes, and the degree of chaos. The standard Gibbs free energy (Δ*G°*, kJ/mol), standard enthalpy (Δ*H*°, kJ/mol), and standard enthalpy (Δ*S°*, J/mol·K) were derived using the following equations [48]:

(7) $$\Delta G{}^{\circ}=-RT\ln K_{e}$$

(8) ΔG°=ΔH°−TΔS°

(9) $$\ln K_{e}=-\frac{\Delta H{}^{\circ}}{RT}+\frac{\Delta S{}^{\circ}}{R}$$

(10) $$K_{e}=\frac{1000K_{L}MC{}^{\circ}}{\gamma }$$

where *R* stands for the universal gas constant (8.314 J/mol·K), *T* is the experimental temperature (K), *K_e_* is a dimensionless parameter that denotes the thermodynamic equilibrium constant, *K_L_* is the Langmuir constant (L/mg), *M* and *C°* are the dye molecular weight (g/mol) and the standard dye concentration (mol/L), respectively, and *γ*, a dimensionless parameter, is the activity coefficient of RY2.

The plot of *lnK_e_* versus 1*/T* determined the parameters as displayed in Table 5. The *ΔG°* values were negative, revealing that the adsorption of RY2 on PEI/APTES-MWCNTs was spontaneous in the temperature range of 15 to 35 °C. In contrast, the positive value of *ΔH°* suggested that the adsorption process was endothermic [49]. The significant and positive value of *ΔS°* indicates that the adsorption of RY2 on PEI/APTES-MWCNTs was an entropy-increasing process with an increase in randomness at the solid–liquid interface during the adsorption process [50]. Overall, the adsorption of RY2 onto PEI/APTES-MWCNTs was favorable at higher temperatures.

### 2.6. Effect of Ionic Strength

Industrial dye wastewater usually contains various ionic salts, typically NaCl [51]. Therefore, NaCl was selected to study the effect of interfering anions and cations on the adsorption of RY2 by PEI/APTES-MWCNTs. The experiment was performed by mixing 30 mL of 200 mg/L RY2 solutions (pH = 2) with 10 mg of adsorbent at 25 °C for 24 h. The NaCl concentrations ranged from 0 to 0.1 mol/L; the results are demonstrated in Figure 8a. With the NaCl concentration increased from 0 to 0.1 mol/L, the adsorption capacity of PEI/APTES-MWCNTs for RY2 only decreased by less than 10%. This phenomenon indicated that cations and anions have little effect on the adsorption of reactive dye molecules on PEI/APTES-MWCNTs.

### 2.7. Reusability Studies

**Figure 9:**
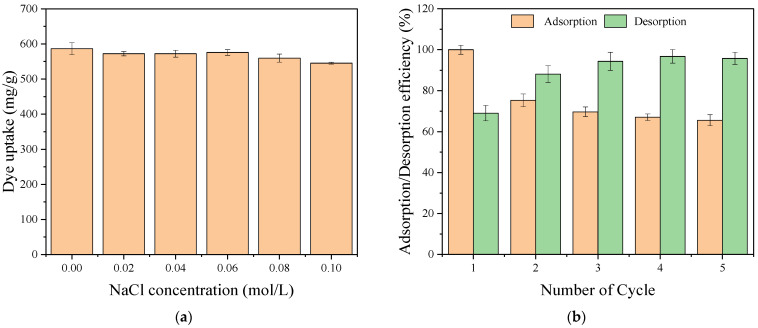
(**a**) Effect of ionic strength on RY2 adsorption by PEI/APTES-MWCNTs; (**b**) adsorption/desorption efficiencies of PEI/APTES-MWCNTs for RY2 removal.

Reusability is an appealing quality of promising adsorbents, as it can reduce investment. The reusability of the PEI/APTES-MWCNTs was verified by conducting the adsorption/desorption cycle five times, and the results are displayed in Figure 9b. The adsorption efficiency remarkably decreased from 100.0 to 75.3% after the first adsorption/desorption cycle. Hereafter, it decreased slightly to 65.6% after five adsorption/desorption cycles. On the contrary, the desorption efficiency increased notably from 69.0% to 94.3% from the first to the third cycles. Then, it remained stable at 96.0 ± 1.0% for the following cycles. Although the adsorption capacity of the adsorbent was lost by about 25% after the first adsorption/desorption cycle, the loss was less in the subsequent cycles and even negligible after the third cycle, so the adsorbent can be considered to have a good potential for industrial applications.

## 3. Materials and Methods

### 3.1. Materials

MWCNTs (diameter: 8–15 nm; length: 5–20 μm; grade: TMC-100-10; purity: >90 wt%) were supplied by Nano Solution Co., Ltd. (Jeonju, Republic of Korea). Branched PEI (M_w_: 70,000, 50% (*w*/*v*) aq. soln.) was obtained from Habjung Moolsan Co., Ltd. (Seoul, Republic of Korea). APTES (99%) was purchased from Daejung Chemical & Metals Co., Ltd. (Siheung, Republic of Korea). Epichlorohydrin (ECH, ≥99%) and RY2 (content: 60~70%) were purchased from Sigma-Aldrich Korea Ltd. (Yongin, Republic of Korea). All of the other chemicals used in this study were of analytical grade.

### 3.2. Preparation of PEI/APTES-MWCNTs

**Figure 10:**
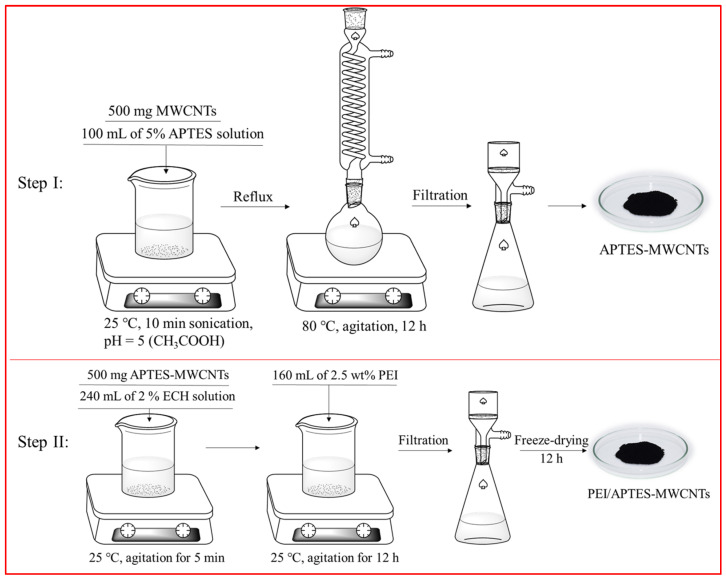
Scheme of the process of PEI/APTES-MWCNTs preparation.

**Figure 11:**
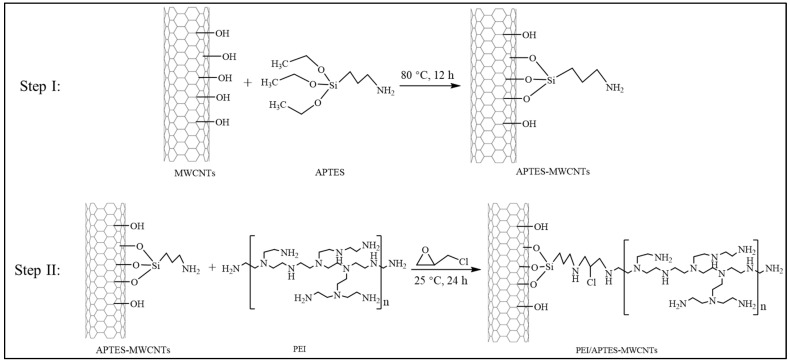
Scheme of the reaction pathway in the preparation of PEI/APTES-MWCNTs.

The preparation of PEI/APTES-MWCNTs consists of two steps: grafting APTES on the MWCNTs’ surface and crosslinking PEI with APTES-MWCNTs using ECH as a crosslinker. The illustrations of the preparation process and the reaction pathway are presented in Figure 10 and Figure 11, respectively. Here are the details:

Step I: APTES-MWCNTs were prepared according to the method of Anton et al. (2020) [52] with slight modifications. Briefly, 1.5 g of MWCNTs was dispersed in 300 mL of a 5% APTES solution (pH = 5, adjusted by CH_3_COOH). The solution was homogenized at 13,500 rpm for 10 min using a WiseTis homogenizer (HG-15A, Witeg, Wertheim, Germany). The mixture was refluxed at 80 °C for 12 h with continuous stirring at 100 rpm. The resulting product (APTES-MWCNTs) was washed with distilled water until pH = 7 and collected by filtration through a 0.22 μm PVDF membrane. One-third of the product (approximately equivalent to 0.5 g MWCNTs) was then taken out, freeze-dried for 24 h, and set aside for further use, while the rest was used in the next step.

Step II: The APTES-MWCNTs prepared in the first step (approximately equivalent to 1.0 g MWCNTs) were mixed with 240 mL of 2% ECH solution and homogenized for 5 min. Then, 160 mL of a 2.5 wt% PEI solution was added to this mixture and homogenized again for 10 min. The mixing solution was stirred continuously at 300 rpm for 12 h at room temperature (25 ± 1 °C). The final product (PEI/APTES-MWCNTs) was filtered and rinsed against distilled water several times until the filtrate was neutral. The PEI/APTES-MWCNTs were dried for 24 h using a freeze dryer and then stored in a desiccator for future experiments.

### 3.3. Characterization of Samples

An attenuated total reflection Fourier transform infrared spectrometer (ATR-FTIR, Nicolet IS50, Thermo Fisher, Waltham, MA, USA) was used to record the FTIR spectra of MWCNTs, APTES-MWCNTs, and PEI/APTES-MWCNTs. The surface morphologies of the materials were observed using FE-SEM (Apreo S, Thermo Fisher, Waltham, MA, USA). The elemental mapping was simultaneously recorded using an EDS (Ultim^®^MAX, Oxford Instruments, Bristol, UK). BET analysis was conducted using a specific surface area analyzer (3 Flex, Micromeritics, Norcross, GA, USA). The zeta potentials of MWCNTs and PEI/APTES-MWCNTs were analyzed using a zeta potential analyzer (ELSZ-2000, Otsuka, Japan).

### 3.4. Adsorption Experiments

The pH edge experiments were performed at an initial dye concentration of 200 mg/L in the pH range of 2–12 for 24 h. The pH was adjusted using 1 M NaOH or 1 M HCl. Adsorption kinetics experiments were conducted at initial dye concentrations of 50, 100, and 200 mg/L at pH 2. Adsorption isotherm experiments were performed at initial dye concentrations ranging from 30 to 500 mg/L at pH 2 for 24 h. The effect of temperature on the adsorption of dyes by PEI/APTES-MWCNTs was also studied at 288, 298, and 308 K. The effect of ionic strength was evaluated by adding a certain amount of NaCl into 30 mL of 200 mg/L RY2 solutions, making the NaCl concentrations range from 0 to 0.1 mol/L. The initial and final dye concentrations were measured using a UV/Vis spectrophotometer (X-ma 3000 pc, Human, Seoul, Republic of Korea). All adsorption experiments were conducted by mixing 10 mg of adsorbent with 30 mL of RY2 solution (pH = 2) in 50 mL Falcon tubes. The tubes were placed in a multi-shaking incubator at 160 rpm and 25 °C. In all adsorption experiments, three parallel samples were prepared for each sample. The dye uptake (*q*, mg/g) was calculated using the equation below:

(11) $$q=\frac{C_{i}V_{i}-C_{f}V_{f}}{m}$$

where *C_i_* and *C_f_* are the initial and final dye concentrations (mg/L), respectively; *V_i_* and *V_f_* are the solution volumes (mL) before and after adsorption experiments; and m is the adsorbent weight (g).

### 3.5. Reusability Experiments

First, RY2-loaded adsorbent was prepared by mixing 10 mg of PEI/APTES-MECNTs with 30 mL of 200 mg/L RY2 solutions for 10 h. The dye-loaded adsorbent was rinsed with distilled water (pH = 2) two times and subjected to desorption. The dye-loaded adsorbent was mixed with 30 mL of alkaline acetone solution (prepared by mixing 0.2 M NaOH and acetone in a volume ratio of 1:1) in 50 mL Falcon tubes and placed in a multi-shaking incubator at 25 °C and 160 rpm for 2 h. After centrifugation, the supernatant was used to measure the concentration of the desorbed dye. The desorbed adsorbent was washed three times with distilled water and then used in the next adsorption–desorption cycle. The regeneration experiments were performed five times in total. The adsorption and desorption efficiencies were calculated using Equations (12) and (13).

(12) $$\mathrm{Adsorption}\;\mathrm{efficiency}\;\left(\%\right)=\frac{\mathrm{Adsorbed}\;\mathrm{dye}\;\mathrm{amount}\;\mathrm{in}\;\mathrm{each}\;\mathrm{cycle}\;\left(\mathrm{mg}\right)}{\mathrm{Original}\;\mathrm{dye}\;\mathrm{adsorption}\;\mathrm{amount}\;\left(\mathrm{mg}\right)}\times 100$$

(13) $$\mathrm{Desorption}\;\mathrm{efficiency}\;\left(\%\right)=\frac{\mathrm{Desorbed}\;\mathrm{dye}\;\mathrm{amount}\;\mathrm{in}\;\mathrm{each}\;\mathrm{cycle}\;\left(\mathrm{mg}\right)}{\mathrm{Adsorbed}\;\mathrm{dye}\;\mathrm{amount}\;\mathrm{in}\;\mathrm{each}\;\mathrm{cycle}\;\left(\mathrm{mg}\right)}\times 100$$

## 4. Conclusions

In this study, we prepared amine-functionalized MWCNTs, namely PEI/APTES-MWCNTs, by a facile method. FE-SEM-EDS, BET, and zeta potential analysis proved that the material was successfully prepared. The adsorption performance of the PEI/APTES-MWCNTs for reactive dyes was evaluated using RY2 as a representative. One-point-check experiment results exhibited that the adsorption capacity of RY2 on PEI/APTES-MWCNTs was 2.8 times higher than that of MWCNTs. Kinetic studies showed that the PEI/APTES-MWCNTs are an effective adsorbent capable of rapidly eliminating reactive dyes from aqueous solutions over a wide range of initial concentrations. Specifically, at an initial concentration of 50–100 mg/L, more than 99% of the dye molecules could be separated from water in less than 15 min. When the initial concentration was increased to 200 mg/L, over 80% of the dye molecules could still be isolated from water within 30 min. Isotherm studies showed that the maximum dye uptake at 15, 25, and 35 °C was 689.66, 714.29, and 735.30 mg/g, respectively. The PSO model better explained the kinetic adsorption process, and the Langmuir model was better suited to explain the adsorption isotherm process. Thermodynamic studies revealed that the adsorption process was spontaneous and endothermic. The effect of ionic strength indicated that the adsorbent has excellent resistance to ionic salt interference. The reusability study revealed that the adsorbent has good reusability. In conclusion, the PEI/APTES-MWCNTs are effective and reusable adsorbents for rapid and highly efficient adsorption of reactive dyes from aqueous solutions.

## Figures and Tables

**Table 1:** Specific surface area, average pore size, and total pore volume of the materials.

Sample	Specific Surface Area (m^2^/g)	Average Pore Size (nm)	Total Pore Volume (cm^3^/g)
MWCNTs	405.22	39.67	4.02
APTES-MWCNTs	289.65	6.50	0.47
PEI/APTES-MWCNTs	176.16	6.30	0.28

**Table 2:** Parameters of pseudo-first-order (PFO), pseudo-second-order (PSO), and intraparticle diffusion (IPD) models.

Models	Parameters	Initial Concentration (mg/L)		
		50	100	200
	*q_e,exp_* (mg/g)	153.03	290.10	576.15
PFO	*q*_1_ (mg/g)	116.68	135.06	152.11
	*k*_1_ (min^−1^)	0.3955	0.1450	0.0083
	*Adj. R* ^2^	0.9804	0.8583	0.5054
PSO	*q*_2_ (mg/g)	163.40	299.40	578.03
	*k*_2_ (g/(mg·min))	0.0057	0.0024	0.0004
	*Adj. R* ^2^	0.9995	0.9998	0.9999
IPD_1st_	*k_i_*_1_ (mg/(g·min^0.5^))	32.269	50.753	80.348
	*C_i_*_1_ (mg/g)	65.06	108.30	201.75
	*Adj. R^2^*	0.9822	0.9686	0.9239
IPD_2st_	*k_i_*_2_ (mg/(g·min^0.5^))	4.028	2.478	7.330
	*C_i_*_2_ (mg/g)	137.43	273.47	466.17
	*Adj. R* ^2^	0.9781	0.9915	0.9852
IPD_3st_	*k_i_*_3_ (mg/(g·min^0.5^))	—	—	1.700
	*C_i_*_3_ (mg/g)			534.05
	*Adj. R* ^2^			0.9368

**Table 3:** Langmuir and Freundlich parameters of RY2 adsorption on PEI/APTES-MWCNTs.

*T* (°C)	*q_exp_* (mg/g)	Langmuir Model				Freundlich Model		
		*q_max_* (mg/g)	*K_L_* (L/mg)	*R_L_*	*Adj. R* ^2^	*K_F_* (mg/g)(L/mg)^1/n^	*n*	*Adj. R* ^2^
15	705.10	689.66	0.6444	0.0001–0.0008	0.9977	432.49	11.17	0.9776
25	725.84	714.29	0.8333		0.9984	446.71	10.79	0.9570
35	742.43	735.30	1.3892		0.9992	475.49	11.05	0.8580

**Table 4:** Comparison of the adsorption capacities of functionalized MWCNTs for anionic dyes.

Adsorbent	Dye	*q_m_* (mg/g)	*T* (°C)	pH	Ref.
PEI/APTES-MWCNTs	Reactive Yellow 2	689.66	15	2	This work
		714.29	25	2	
		735.29	35	2	
MWCNTs/Gly/β-CD	Acid Blue 113	172.41	25	7	[43]
	Methyl Orange	96.15	25	5	
	Disperse Red 1	500	25	7	
MWCNTs-NH_2_	Acid Black 1	666	25	2	[44]
	Acid Black 25	714	25	2	
NH_2_-MWCNTs	Methyl Orange	185.53	25	2	[45]
MnFe_2_O_4_/MWCNTs	Direct Red 16	607.79	55	2	[46]
MWCNTs-UiO-66	Methyl Red	105.26	25	3.61	[47]
MWCNTs/Fe_3_O_4_@(PDA + PEI)	Methyl Orange	935	25	7	[14]
	Congo Red	1006	25	7	

**Table 5:** Thermodynamic parameters of RY2 adsorption on PEI/APTES-MWCNTs.

*T* (°C)	*K_e_*	Δ*G* (kJ/mol)	Δ*H°* (kJ/mol)	Δ*S°* (J/mol·K)	*Adj. R* ^2^
15	5.36 × 10^5^	−31.58	28.21	207.27	0.9147
25	6.92 × 10^5^	−33.32			
35	11.54 × 10^5^	−35.75			

## Data Availability

All data generated in this study are presented in the current manuscript. Data are available upon request from the corresponding author.
